# Outcomes of ulna shortening osteotomy: a cohort analysis of 106 patients

**DOI:** 10.1186/s10195-021-00621-8

**Published:** 2022-01-05

**Authors:** J. S. Teunissen, R. M. Wouters, S. Al Shaer, O. T. Zöphel, G. M. Vermeulen, S. E. R. Hovius, E. P. A. Van der Heijden, The Hand-Wrist Study Group, R. A. M. Blomme, B. J. R. Sluijter, D. J. J. C. van der Avoort, A. Kroeze, J. Smit, J. Debeij, E. T. Walbeehm, G. M. van Couwelaar, G. M. Vermeulen, J. P. de Schipper, J. F. M. Temming, J. H. van Uchelen, H. L. de Boer, K. P. de Haas, K Harmsen, O. T. Zöphel, R. Feitz, G. J. Halbesma, J. S. Souer, R. Koch, S. E. R. Hovius, T. M. Moojen, X. Smit, R. van Huis, P. Y. Pennehouat, K. Schoneveld, Y. E. van Kooij, R. M. Wouters, J. J. Veltkamp, A. Fink, W. A. de Ridder, H. P. Slijper, R. W. Selles, J. T. Porsius, J. Tsehaie, R. Poelstra, M. C. Jansen, M. J. W. van der Oest, P. O. Sun, L. Hoogendam, J. S. Teunissen, Jak Dekker, M. Jansen-Landheer, M. ter Stege, J. M. Zuidam, J. W. Colaris, L. Duraku, E. P. A. van der Heijden, D. E. van Groeninghen

**Affiliations:** 1 Department of Plastic Surgery, Radboud University Medical Centre, Geert Grooteplein Zuid 10, 6525 Nijmegen, GA The Netherlands; 2 Hand and Wrist Centre, Xpert Clinic, Amsterdam, The Netherlands; 3 Department of Plastic, Reconstructive and Hand Surgery, Erasmus MC, University Medical Centre, Rotterdam, The Netherlands; 4 Department of Plastic Surgery, Ziekenhuisgroep Twente, Almelo, The Netherlands; 5 Department of Rehabilitation Medicine, Erasmus MC, University Medical Centre Rotterdam, Rotterdam, The Netherlands; 6 Department of Plastic Surgery, Jeroen Bosch Ziekenhuis, ‘S-Hertogenbosch, The Netherlands; 7 Nijmegen, The Netherlands

**Keywords:** Ulna shortening osteotomy, Ulnar impaction syndrome, DRUJ, DRF, PROM

## Abstract

**Background:**

Ulna shortening osteotomy (USO) for ulnar impaction syndrome (UIS) aims to improve pain and function by unloading the ulnar carpus. Previous studies often lack validated patient-reported outcomes or have small sample sizes. The primary objective of this study was to investigate patient-reported pain and hand function at 12 months after USO for UIS. Secondary objectives were to investigate the active range of motion, grip strength, complications, and whether outcomes differed based on etiology.

**Materials and methods:**

We report on 106 patients with UIS who received USO between 2012 and 2019. In 44 of these patients, USO was performed secondary to distal radius fracture. Pain and function were measured with the Patient Rated Wrist/Hand Evaluation (PRWHE) before surgery and at 3 and 12 months after surgery. Active range of motion and grip strength were measured before surgery and at 3 and 12 months after surgery. Complications were scored using the International Consortium for Health Outcome Measurement Complications in Hand and Wrist conditions (ICHAW) tool.

**Results:**

The PRWHE total score improved from a mean of 64 (SD = 18) before surgery to 40 (22) at 3 months and 32 (23) at 12 months after surgery (*P*  < 0.001; effect size Cohen’s *d* = −1.4). There was no difference in the improvement in PRWHE total score (*P* = 0.99) based on etiology. Also, no clinically relevant changes in the active range of motion were measured. Independent of etiology, mean grip strength improved from 24 (11) before surgery to 30 (12) at 12 months (*P*  = 0.001). Sixty-four percent of patients experienced at least one complication, ranging from minor to severe. Of the 80 complications in total, 50 patients (47%) had complaints of hardware irritation, of which 34 (32%) had their hardware removed. Six patients (6%) needed refixation because of nonunion.

**Conclusion:**

We found beneficial outcomes in patients with UIS that underwent USO, although there was a large variance in the outcome and a relatively high number of complications (which includes plate removals). Results of this study may be used in preoperative counseling and shared decision-making when considering USO.

**Level of evidence:**

Therapeutic III.

**Supplementary Information:**

The online version contains supplementary material available at 10.1186/s10195-021-00621-8.

## Introduction

Ulnar impaction syndrome (UIS) is a condition at the ulnar side of the wrist that occurs because of continuous or intermittent chronic excessive loading across the ulnocarpal joint [1]. It occurs mainly in patients with positive ulnar variance. Palmer showed that an increase of the ulnar length by 2.5 mm increases the ulnar load by 42% [2]. Patients with UIS may suffer from symptoms such as ulnar-sided wrist pain, decreased range of motion, impaired grip strength, and limitations in daily living [1, 3]. Most patients with UIS start with nonoperative management such as nonsteroidal antiinflammatory drugs (NSAIDs), orthoses, corticoid injections, and hand therapy. When nonoperative management is insufficiently effective, surgical treatment can be considered.

Ulna shortening osteotomy (USO) aims to decompress the ulnar load and is a frequently used surgical treatment for patients with UIS [4, 5]. However, only a few studies with a low sample size of 10–20 patients have evaluated the effectiveness of USO using validated and reliable patient-reported outcome measures (PROMs) [6–9]. More studies with larger sample sizes are needed to validate the results of these studies. Furthermore, the influence of UIS etiology (e.g., idiopathic UIS versus UIS secondary to distal radius fracture) on treatment outcomes is unclear.

Previous studies on USO also described the complications following USO [10–12], including nonunion or the need for plate removal due to irritation. Chan et al. [10] summarized the prevalence of complications across studies and found large variations, e.g., plate removal ranged from 0% to 45%. Furthermore, they compared their patients with previous literature and found higher complication rates, suggesting that complications after USO may not be systematically registered using a standardized tool such as the recently developed International Consortium for Health Outcome Measurement Complications in Hand and Wrist conditions (ICHAW).

The primary objective of this study was to investigate the patient-reported pain and hand function at 12 months after USO for UIS. Secondary objectives were to investigate the active range of motion, grip strength, complications, and whether outcomes differed based on etiology.

## Patients and methods

### Study design and setting

We conducted a study involving prospectively gathered data on a consecutive cohort of patients that underwent USO between January 2012 and October 2019 at Xpert Clinics, The Netherlands. All hand surgeons at our institution are certified by the Federation of European Societies for Surgery of the Hand and over 150 hand therapists.

All patients who underwent USO were invited to be part of a routine outcome measurement system after their first consultation with a hand surgeon. Upon agreement, they received secure web-based questionnaires before and at 3 and 12 months after surgery using GemsTracker [13]. The exact research setting of our study group has been reported previously [14].

We report this study using the Strengthening the Reporting of Observational Studies in Epidemiology (STROBE) statement [15]. The Ethics Committee of the Erasmus University Medical Centre approved the study protocol. All patients provided written informed consent for their data to be anonymously used in this study.

### Participants

**Fig. 1:**
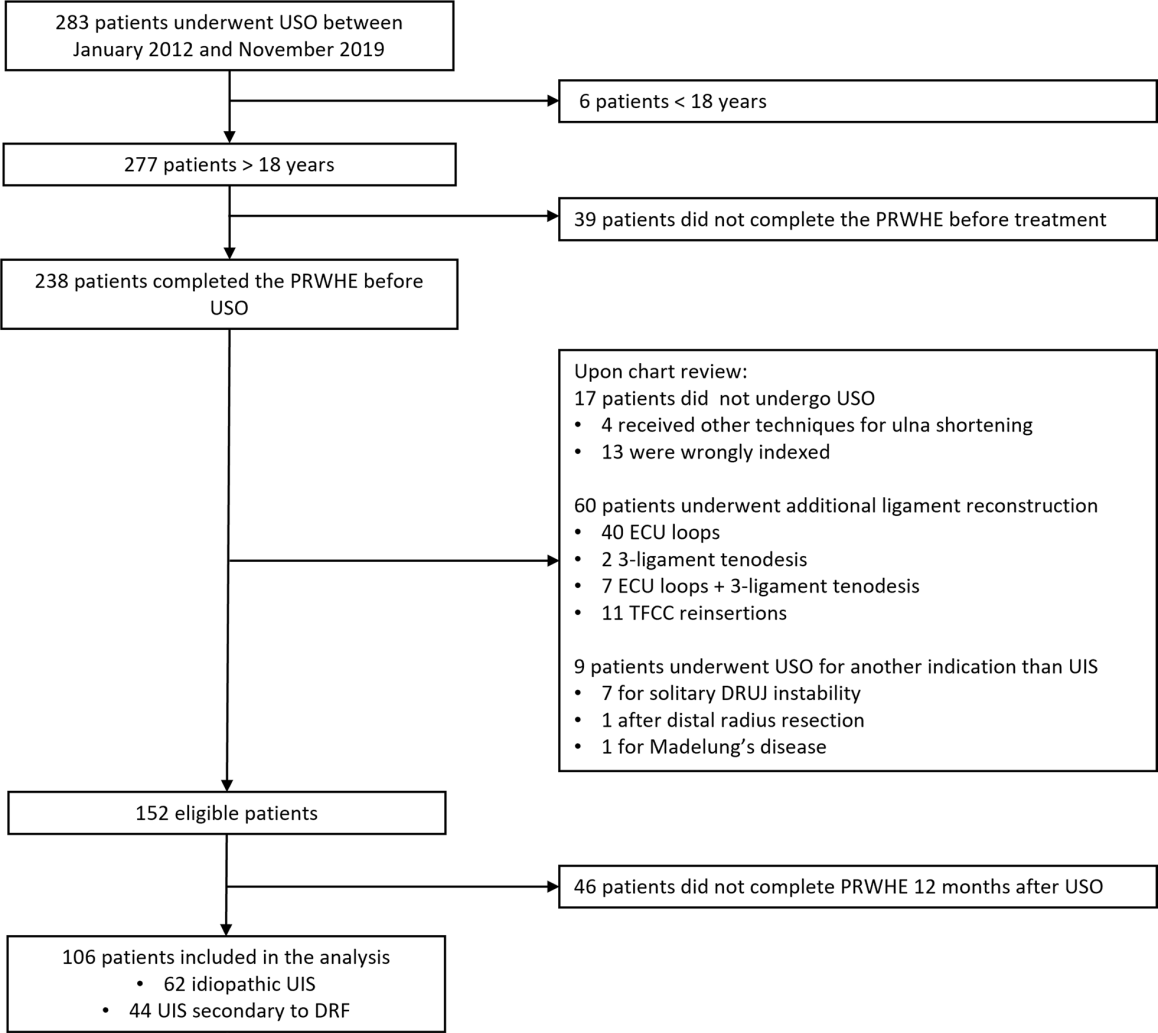
Flowchart of the study. *USO* ulna shortening osteotomy, *PRWHE* Patient Rated Wrist/(Hand) Questionnaire, *ECU* extensor carpi ulnaris, *TFCC* triangular fibrocartilage complex, *DRUJ* distal radioulnar joint, *DRF* distal radius fracture

A total of 283 patients underwent ulna shortening osteotomy during the study period. We excluded 6 patients that were younger than 18 years and 39 patients who did not complete the questionnaires before surgery. We reviewed electronic patient records of the remaining 238 patients to confirm that USO was performed for UIS, as USO may also be used for other indications. To be classified as UIS, at least one of the following criteria needed to be met: (1) the surgeons explicitly diagnosed the patients with UIS in the electronic patient records; (2) wrist arthroscopy showed signs of Palmer type 2 lesions, such as Triangular Fibrocartilage Complex (TFCC) degeneration and lunate chondropathy, [16]; (3) magnetic resonance imaging (MRI) showed signs of focal abnormal signal intensity in the lunate, triquetrum, and ulnar head [17]; (4) there was evident ulnar positive variance on standard posterior–anterior wrist radiographs in a neutral position [18]. This definition excluded patients that underwent USO for other indications, such as solitary distal radioulnar joint (DRUJ) instability or Madelung’s disease. Patients who underwent simultaneous ligament reconstruction for instability [extensor carpi ulnaris (ECU) loop, 3-ligament tenodesis, and TFCC reinsertion] were also excluded. This left 155 patients, of which we included 106 patients who completed all questionnaires after 12 months. Furthermore, we classified patients as having UIS secondary to distal radius fracture malunion or idiopathic UIS. The flowchart of the patient inclusion is shown in Fig. 1.

### Surgical procedure and rehabilitation

Surgery was performed under general anesthesia and/or a regional axillary or supraclavicular block by 13 hand surgeons. A longitudinal incision was made on the ulnar surface and the ulna was exposed between the flexor carpi ulnaris and extensor carpi ulnaris. Care was taken not to damage the dorsal sensory branch of the ulnar nerve. The osteotomy was performed at the level of the diaphysis using a freehand cut or an external cutting device based on the surgeon’s preference, and the ulna was shortened by several millimeters, depending on the amount of preoperative radioulnar variance. The ulna was fixated using a plate on the volar or dorsal surface on the ulna based on the surgeon’s preference (*n* = 55 Acumed, Hillsboro, Oregon, USA; *n* = 47 AO, Davos, Switzerland, *n* = 1 Recos KLS Martin, Tuttlingen, Germany, *n* = 1 Trimed, Santa Clarita, California, USA, *n* = 1 Zimmer Biomet, Dordrecht, the Netherlands, *n* = 1 Medartis, Basel, Switzerland). The skin was closed with Monocryl or Prolene (Ethicon). The experience of the surgeon was defined following the classification by Tang and Giddins [19].

The routine postoperative immobilization protocol consisted of plaster cast (including the elbow) immobilization for 10–12 days (since 2015 this was reduced to 3–5 days) followed by thermoplastic orthosis until 6 weeks postoperatively. Wrist flexion/extension exercises were initiated 2 weeks postoperatively. Pronation/supination and strengthening exercises were initiated at 6 weeks postoperatively. All patients were encouraged to follow an extensive rehabilitation program including hand therapy exercises. The entire postoperative protocol is shown in the additional file 1: Table S1. Our center for hand surgery and therapy is fully integrated and postoperative hand therapy was closely monitored. Standard radiographs were taken at 3 and 12 months postoperatively to assess bony union, and additional radiographs were made on indication (e.g., in case of delayed union, nonunion, or trauma).

Implant removal is not routinely performed in the Netherlands but may be indicated on clinician-based arguments or patient-based symptoms [20]. Patient-based symptoms are considered a valid reason for hardware removal [21]. Plate removal was considered when patients experienced irritation from the plate following full consolidation on the x-ray.

### Variables and data sources/measurements

Demographic variables that were routinely collected included age, sex, type of work, symptom duration, treatment side, hand dominance, and the smoking status at the time of surgery. We reviewed the medical records to collect data on treatment of the initial injury, operative variables (such as the type and positioning of the fixation plate), and the occurrence of complications.

Patients completed the Dutch-language version of the patient rated wrist/hand evaluation (PRWHE) before surgery and at 3 and 12 months after surgery [22]. Previous research found that it is a very responsive patient-derived questionnaire to evaluate the treatment outcomes of UIS [23–25]. The minimal clinically important difference (MCID) in the PRWHE total score for patients who underwent USO for idiopathic UIS is 17 [26].

A hand therapist measured active range of motion (ROM) and grip strength before surgery and at 3 and 12 months after surgery. In this standardized examination following ICHOM guidelines [27], the ROM was measured in degrees from neutral using a goniometer. The goniometer was placed at the dorsal side of the wrist to measure wrist flexion/extension, radial/ulnar deviation, and pronation, and at the volar side of the wrist to measure supination. Wrist flexion, radial deviation, and pronation are reported as positive values; wrist extension, ulnar deviation, and supination as negative values. Grip strength was measured using an E-LINK Jamar-Style dynamometer (Biometrics, Newport, UK) following the methods of Mathiowetz et al. [28].

Complications were scored following the International Consortium for Health Outcome Measurement (ICHOM) Complications in Hand and Wrist conditions (ICHAW) classification, which is modified from the Clavien–Dindo classification for general surgery (see additional file 2: Table S2) [29]. This tool classifies complications within 12 months after surgery into different grades based on the treatment it requires. When a complication is not sufficiently relieved with minimally invasive treatment and more invasive treatment was given, only the complication with the highest grade is reported.

The primary outcome of this study was the change in PRWHE total score at 12 months after surgery. Secondary outcomes were complications, ROM, and grip strength.

### Statistical analysis and study size

We performed a post hoc power analysis, with a conventional effect size of 0.3, *α* error probability of 0.05, and a sample size of 106 patients, and achieved a power of 92%.

We checked continuous data for normal distributions with histograms and quantile–quantile plots. Normally distributed data were displayed as mean values including standard deviations (SD) and skewed data were displayed as mean values including interquartile ranges (IQR). We used linear mixed models to compare data with more time points. We calculated the effect size of Cohen’s *d* between preoperative and 12 months PRWHE scores [30]. We compared continuous data between groups using independent *T* tests or Mann–Whitney *U* tests, and categorical data using chi-squared tests.

Because data were collected during daily clinical practice, missing data were expected in the PRWHE score at 12 months follow-up. We performed Little’s test to investigate whether the PRWHE scores at 12 months after surgery were missing completely at random [31]. Furthermore, we tested for significant differences in demographics and preoperative scores between patients who completed the PRWHE before and at 12 months after surgery (defined as responders) and patients who did not fill in the PRWHE at both time points (defined as nonresponders).

All computations were performed in R v4.0.1 (R Project for Statistical Computing, Vienna, Austria). A *P* value  < 0.05 was considered significant.

## Results

### Demographics of the study population

Table 1 presents the demographics, surgical specifics, and preoperative measurements. The mean age of the study patients was 50 (standard deviation:  ±11) years and 32% of the patients were males. In 42% of the patients, the UIS was secondary to distal radius fracture. Twelve patients had previously undergone a corrective osteotomy of the distal radius. Compared with the idiopathic UIS group, patients with UIS secondary to distal radius fracture were older (*P* = 0.044), had less range of motion in all directions except radial deviation (*P* < 0.001–0.012), had less grip strength (*P* = 0.008) at baseline, and had more millimeters resected during the USO (*P* < 0.001). Little’s test (*P* = 0.79) and the nonresponder analysis (additional file 3: Table S3) suggested that missing data on PRWHE at 12 months were missing completely at random.

**Table 1:** Characteristics of the study population

Characteristic	Overall	Idiopathic	Secondary to DRF	*P*-value
*n*	106	62	44	
Age, mean (SD) (in years)	50 (11)	48 (11)	52 (11)	0.044
Sex = Male, *n* (%)	32 (30)	19 (31)	13 (30)	1.000
Duration of symptoms, median [IQR]	12 [8, 30]	18 [9, 36]	12 [7, 24]	0.089
Type of work, *n* (%)				0.605
None	32 (30)	17 (27)	15 (34)	
Light	24 (23)	14 (23)	10 (23)	
Medium	32 (30)	18 (29)	14 (32)	
Heavy	18 (17)	13 (21)	5 (11)	
Dominant side affected = No, *n* (%)	47 (44)	25 (40)	22 (50)	0.430
Smoker, *n* (%)				0.421
Yes	22 (21)	15 (24)	7 (16)	
No	81 (76)	46 (74)	35 (80)	
Unknown	3 (3)	1 (2)	2 (5)	
Preoperative PRWHE, mean (SD)				
Total score	64 (18)	66 (17)	61 (20)	0.195
Pain score	34 (9)	34 (8)	32 (10)	0.240
Function score	61 (21)	63 (20)	58 (23)	0.210
Preoperative active ROM^a^, mean (SD)				
Wrist extension	−56 (14)	−60 (12)	−51 (15)	0.001
Wrist flexion	52 (17)	57 (16)	46 (18)	0.001
Ulnar deviation	−23 (9)	−25 (9)	−21 (8)	0.012
Radial deviation	18 (6)	18 (6)	16 (6)	0.108
Supination	−69 (17)	−72 (13)	−63 (20)	0.006
Pronation	74 (13)	77 (11)	69 (15)	0.003
Preoperative grip strength^b^, mean (SD)	24 (11)	27 (10)	20 (11)	0.008
Ulna shortening^c^ (mm), median [IQR]	4 [3, 4]	3 [3, 4]	4 [3, 4]	< 0.001
Intervention = Concomitant^d^, *n* (%)	18 (17)	6 (10)	12 (27)	0.034

The *P* value is calculated between the groups based on etiology

*SD* standard deviation, *IQR* interquartile range, *DRF* distal radius fracture, *PRWHE* Patient Rated Wrist/Hand Questionnaire, *ROM* range of motion

^a^2% missing data

^b^13% missing data

^c^7% missing data

^d^Carpal tunnel release (*n* = 1); trigger finger release (*n* = 2); posterior interosseous nerve neurectomy (*n* = 2); pisiformectomy (*n* = 3); removal of hardware for distal radius fracture (*n* = 8); wafer (*n* = 1)

### Patient-reported pain and function

**Fig. 2:**
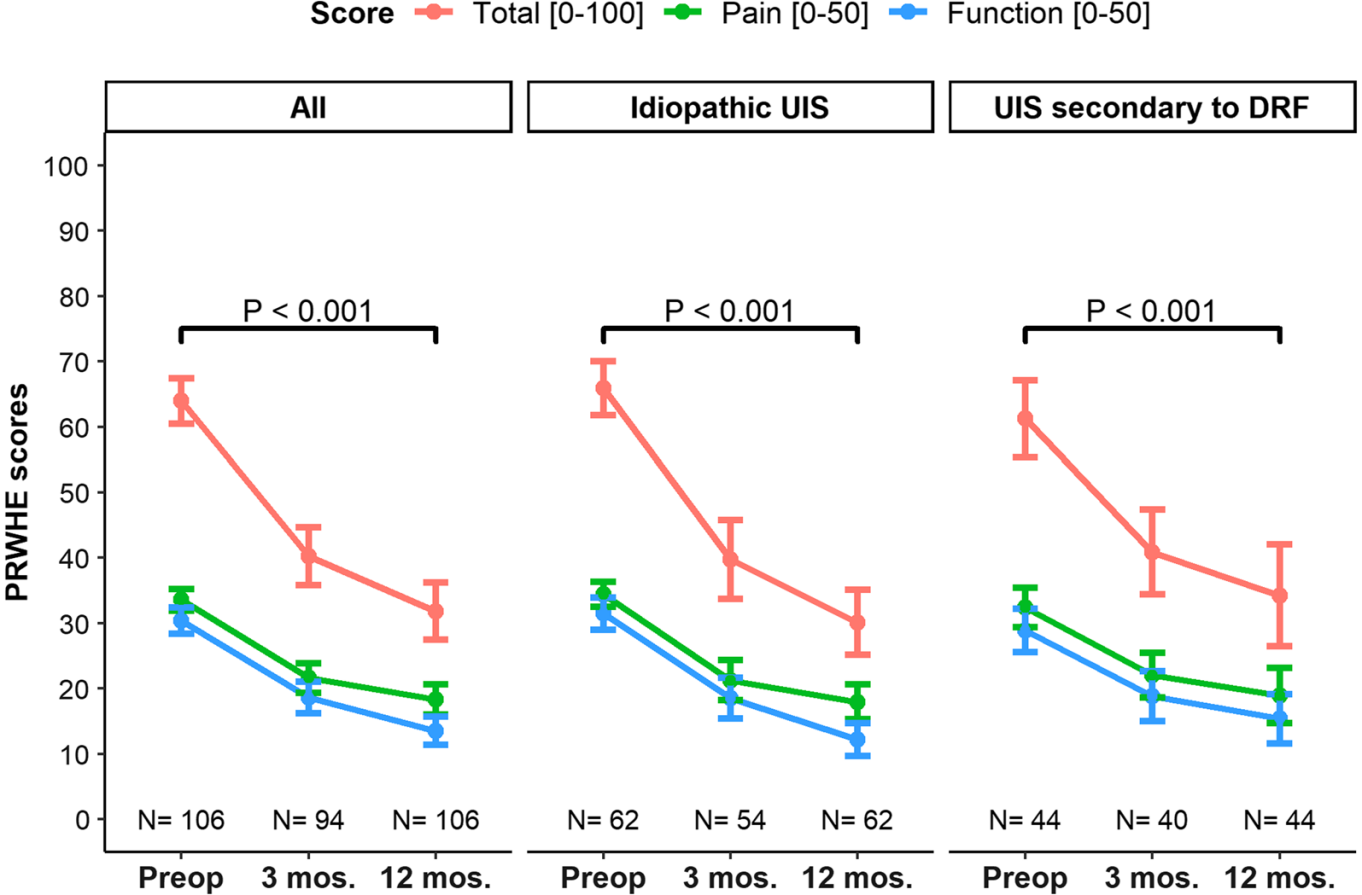
The mean patient rated wrist/hand evaluation total score and subscores before ulna shortening osteotomy and at 3 and 12 months postoperatively. The error bars indicate standard errors. The *P* values indicate significance over time, i.e., whether differences between baseline and follow-up were significant

**Fig. 3:**
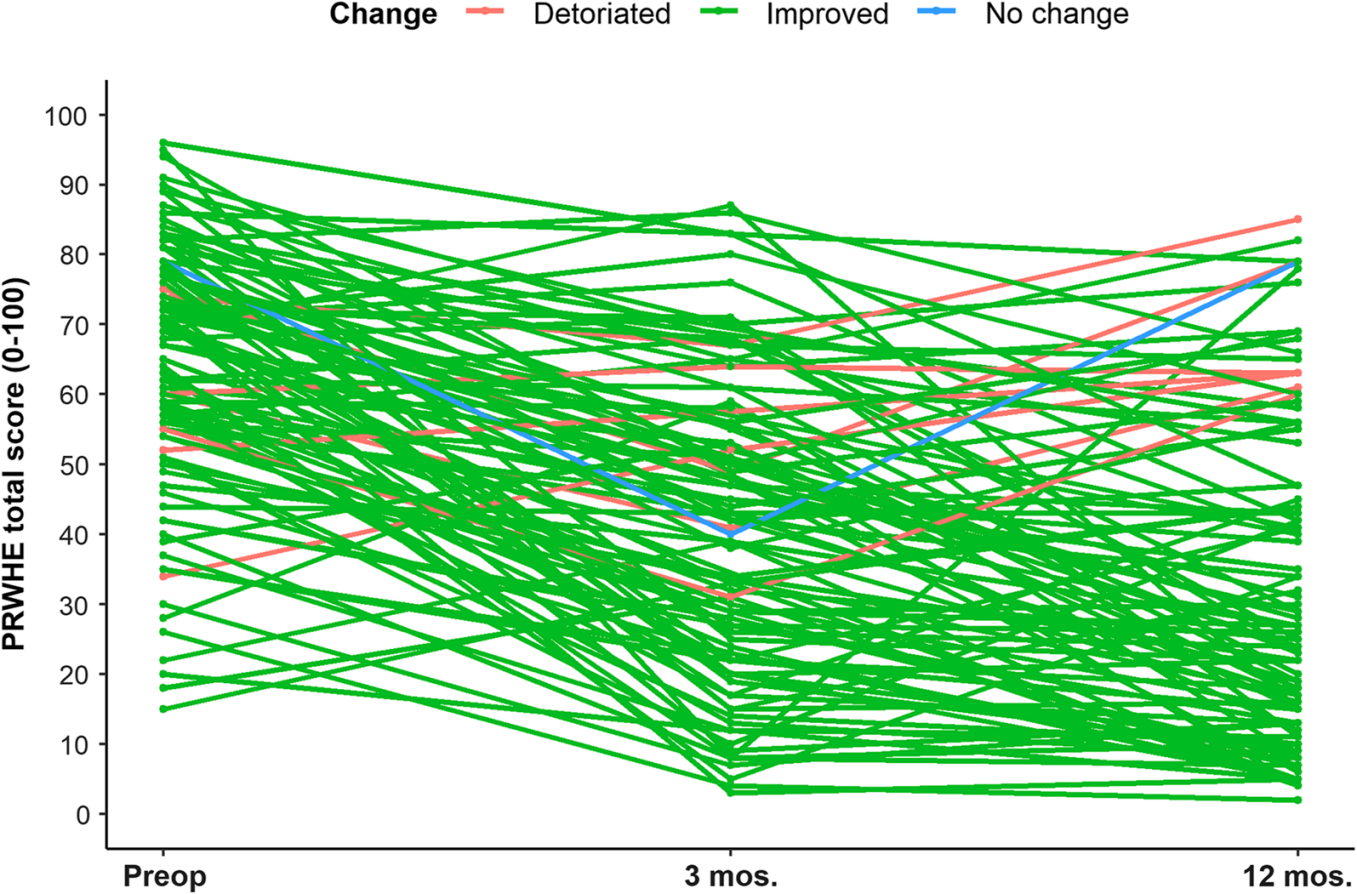
The patient rated wrist/hand evaluation total score before ulna shortening osteotomy and at 3 and 12 months postoperatively plotted for each patient

The PRWHE total score improved from a mean score of 64 (SD = 18) before surgery to 40 (22) at 3 months and 32 (23) at 12 months after surgery (*P* < 0.001; *d*  = −1.4; Fig. 2). Although there was an overall improvement, a large variation in outcomes was observed at all time points (Fig. 3). The PRWHE pain score improved from 34 (9) to 18 (12) at 12 months (*P* < 0.001; *d* = −1.2), and the function score improved from 30 (10) to 14 (11) (*P* < 0.001; *d* = −1.4). There was no difference in the improvement in PRWHE total score (*P* = 0.99), pain score (0.894), or function score (*P* = 0.891) based on etiology.

### Active range of motion and grip strength

**Fig. 4:**
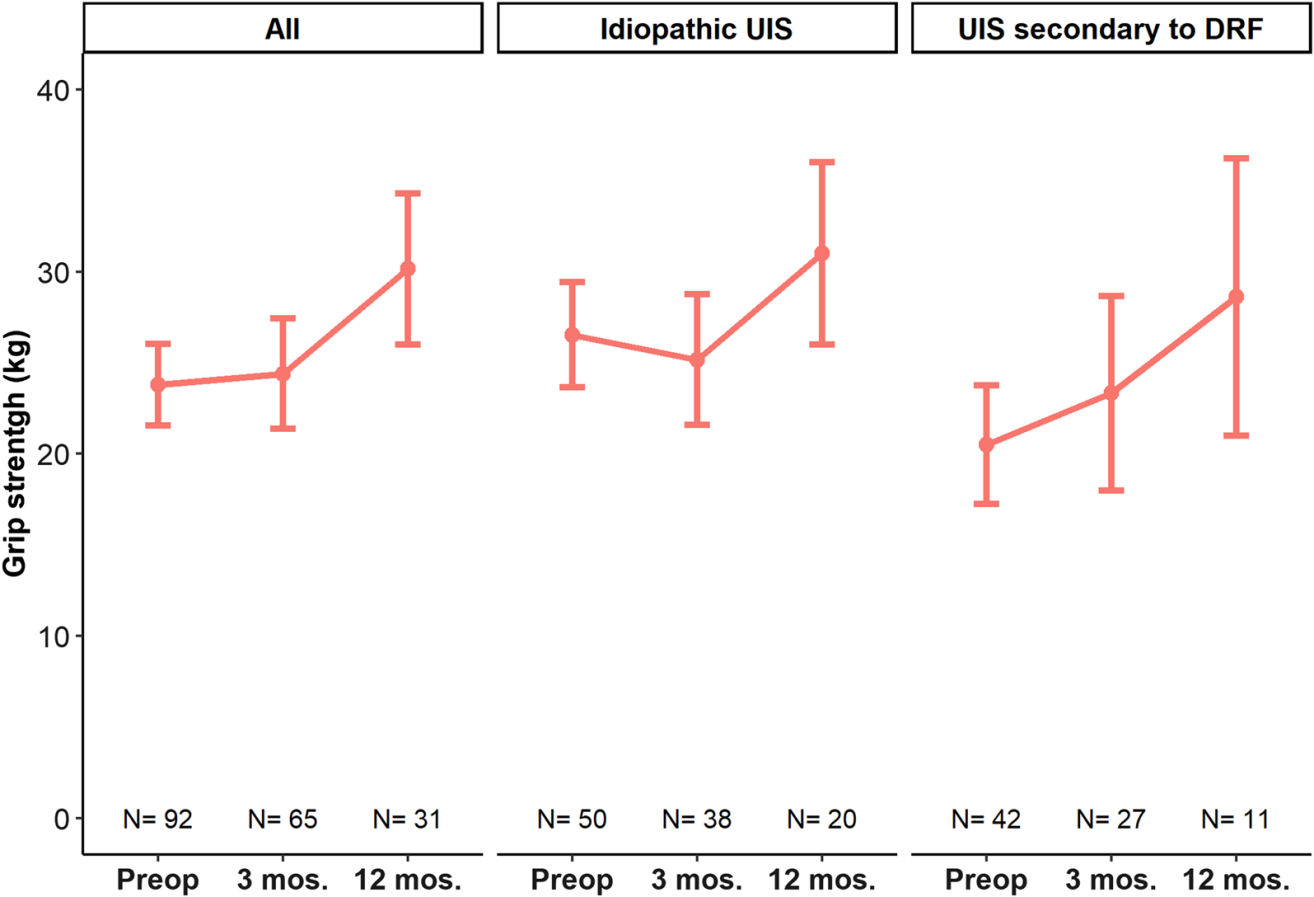
The mean grip strength (kg) before ulna shortening osteotomy and at 3 and 12 months postoperatively. The error bars indicate standard errors

Table 2 presents the range of motion at all time points. Wrist extension improved in all patients, whereas wrist flexion, ulnar deviation, and radial deviation improved only in patients with secondary UIS. The overall mean grip strength improved from 24 (11) before surgery to 30 (12) at 12 months (*P* = 0.001), improvement was seen for both etiologies (*P* idiopathic = 0.006 and *P* secondary to DRF = 0.011) (Fig. 4).

**Table 2:** Range of motion before ulna shortening osteotomy and at 3 and 12 months postoperatively

Group	Movement, mean (SD)	Preoperative	3 months	12 months	*P*-value*
Overall	Wrist extension	−56 (14)	−58 (12)	−64 (8)	< 0.001
	Wrist flexion	52 (17)	52 (12)	60 (12)	0.002
	Ulnar deviation	−23 (9)	−23 (7)	−27 (8)	0.017
	Radial deviation	18 (6)	17 (7)	20 (9)	0.002
	Pronation	−74 (13)	−71 (13)	−74 (11)	0.656
	Supination	69 (17)	65 (15)	70 (13)	0.835
Idiopathic	Wrist extension	−60 (12)	−59 (10)	−64 (8)	0.022
	Wrist flexion	57 (16)	53 (12)	60 (12)	0.062
	Ulnar deviation	−25 (9)	−24 (7)	−27 (7)	0.175
	Radial deviation	18 (6)	19 (8)	21 (10)	0.078
	Pronation	−77 (11)	−74 (12)	−74 (9)	0.218
	Supination	72 (13)	67 (15)	69 (13)	0.260
Secondary to DRF	Wrist extension	−51 (15)	−57 (14)	−65 (10)	0.002
	Wrist flexion	46 (18)	51 (13)	59 (12)	0.021
	Ulnar deviation	−21 (8)	−22 (6)	−27 (9)	0.035
	Radial deviation	16 (6)	15 (6)	20 (9)	0.003
	Pronation	−69 (15)	−68 (14)	−72 (15)	0.682
	Supination	63 (20)	63 (16)	73 (13)	0.151

There were 104 preoperative patients (idiopathic = 61; DRF = 43), 67 at 3 months (idiopathic = 37; DRF = 30), and 29 at 12 months (idiopathic = 19; DRF = 10)

*DRF* distal radius fracture

*P values indicate significance over time, i.e., whether differences between baseline and follow-up were significant

### Complications

Table 3 presents all complications. Sixty-four percent of all patients experienced at least one complication. Of the 80 complications, 50 (47%) were directly related to hardware irritation, 34 of which (32%) had their hardware removed. There were no refractures after plate removal. Six patients (6%) needed refixation because of nonunion; the characteristics of these patients are presented in Table 4. Five patients (5%) had subsequent therapy for persistent ulnar-sided wrist pain, two underwent hand therapy and/or splinting, one underwent TFCC reinsertion, one underwent pisiformectomy, and one underwent neurolysis.

**Table 3:** Complications and reoperations within 12 months after ulna shortening osteotomy

Complication	*n*
No complication	38 (36% had no complications)
Grade I	29 complications in 29 patients (27% had a Grade I complication)
Postoperative bleeding	1
Scar tenderness	1
Hardware irritation	16
Hand therapy	
ECU luxation	1
DRUJ instability	1
Midcarpal laxity	1
Radial tunnel syndrome	1
Persistent ulnar sided wrist pain	1
Splinting	
ECU tendinitis	1
Impaired pronation	1
Persistent ulnar-sided wrist pain	2
Delayed union needing bone stimulation	2
Grade II	Three complications in three patients (3% of the patients had a Grade II complication)
Corticosteroid injection	
Trigger finger	3
Grade IIIA	0 complications (% had a Grade IIIA complication)
Grade IIIB	48 complications in 39 patients (37% had a Grade IIIC complication)
Refixation after nonunion	6
Hardware removal	34
Persistent ulnar-sided wrist pain	
TFCC reinsertion	1
Pisiformectomy	1
Neurolysis	1
3-LT tenodesis	1
Tenolysis	4
Grade IIIC	0 complications

*TFCC* triangular fibrocartilage complex

**Table 4:** Patient and surgical characteristics of the patients that required bone stimulation and/or refixation for delayed union/nonunion

Characteristic	Pt. 1	Pt. 2	Pt. 3	Pt. 4	Pt. 5	Pt. 6	Pt. 7	Pt. 8
Age (years)	46	71	35	48	63	53	41	46
Sex	Female	Female	Male	Female	Male	Male	Female	Female
Duration of symptoms (months)	5	12	10	60	5	24	18	9
Type of work	Heavy	None	Heavy	Medium	None	Heavy	Medium	Medium
Side	Dominant	Nondominant	Dominant	Dominant	Dominant	Nondominant	Dominant	Dominant
Smoking status	No	No	No	No	No	No	No	No
Etiology	DRF	DRF	DRF	Idiopathic	Idiopathic	Idiopathic	Idiopathic	Idiopathic
Plate	Acumed	AO	AO	Acumed	AO	Acumed	Acumed	Acumed
Shortening (mm)	3, 5	4	4, 5	3	4	3	3	^a^
Traumatic injury after USO	No	No	No	No	No	No	Yes	Yes
Bone stimulator (IGEA) used	No	No	No	No	No	Yes	Yes	Yes
Time to revisions surgery (days)	126	119	233 (patient was too busy with work)	143	173	221	NA^b^	NA^b^
Experience level of surgeon^c^	III	IV	III	III	III	III	IV	III

*USO* ulna shortening osteotomy, *DRF* distal radius fracture

^a^Missing

^*b*^*NA* not applicable; union achieved with bone stimulation and refixation not needed

^c^According to the classification by Tang and Giddins (I Non-specialist; II Specialist - less experienced; III Specialist - experienced; IV Specialist - highly experienced; V Expert)

## Discussion

Ulnar impaction syndrome (UIS) is a condition at the ulnar side of the wrist that occurs because of chronic excessive loading across the ulnocarpal joint [1]. Ulna shortening osteotomy (USO) is a frequently used surgical treatment for patients with UIS [4, 5]. In this study, we report on the outcomes of USO using prospectively gathered and reliable patient-reported outcome measures (PROMs) in a relatively large sample size [6–9]. We found that patients with UIS reported less pain and improved function at 12 months after USO. However, there was a large variance in the outcome and a relatively high number of complications, ranging from minor to severe (which includes plate removal). Results of this study may be used in preoperative counseling and shared decision-making when considering USO.

Our study had several limitations. First, there were missing data in the patient and clinician-reported outcomes, making our findings not generalizable to the entire cohort. However, the data were missing at random and there were no baseline differences between responders and nonresponders. Thus, we are confident that the missing data did not influence our findings. A second limitation is that in several electronic patient dossiers, the indication for USO was not explicitly stated. Therefore, we had to categorize these patients retrospectively. Third, the study sample was not homogeneous regarding some factors that may influence the outcome of surgery. While all USOs were performed at the level of the diaphysis using an oblique cut, there was variation in the manner of the osteotomy (freehand versus specific USO devices) and the type and position of the fixation plate, which may have influenced the outcomes during follow-up. Although previous research did not find a difference in pain relief or return to work between freehand USO and specific USO devices [32, 33]. Fourth, some patients underwent concomitant surgery during the USO, which could have induced some co-treatment bias.

Previous studies have reported an overall improvement in patient-reported pain and function after USO in patients with UIS [6–9]. Our data are in line with previous studies and demonstrate improvement following USO in a relatively large sample size. USO can be considered an effective treatment for patients with UIS in general, but it should be noted that we observed a large variation in the patient-reported outcome at 12 months. Some patients remained impaired, and a large prevalence of complications occurred, ranging from minor to severe. The reason for the variation in the patient-reported outcome will be a focus of future research. We found mean improvement for various measures of range of motion over time. This improvement will probably not be clinically relevant as it is of the same magnitude as the measurement error of the goniometer [34]. However, the gain in patient-reported outcomes was not at the cost of the range of motion. This finding is in line with previous research [9, 35, 36]. The grip strength also improved over time.

The scoring of complications after USO following the International Consortium for Health Outcome Measurement Complications in Hand and Wrist conditions (ICHAW) is new. This system, with well-described definitions of complications, was designed to improve the standardization and transparency of complication registration after hand and wrist surgery. Six percent of the patients required refixation with bone graft for radiographically established nonunion. This finding is similar to the results of the meta-analysis reporting nonunion rates after oblique USO [37]. Little is known on the risk factors for nonunion after USO, as the complication is relatively infrequent and most studies on USO (including this one) lack power for statistical inference. Cha found that smoking, low bone density, and decreased range of motion were independently associated with nonunion after USO [38]. Interestingly, all our patients did not smoke at the time of the USO. Many other factors, such as the type of osteosynthesis material, experience of the surgeon, and comorbidities, may lead to an increased risk of nonunion. Our descriptive data may contribute to future meta-analyses on this topic.

Furthermore, 32% of the patients underwent subsequent surgery to remove the plate within 12 months after surgery. This number is expected to increase when applying longer follow-ups. Previous studies have also reported high rates of plate removal of, e.g., 19–43% [10, 39], and in other studies, the plate is routinely removed [40, 41]. Patients should therefore be informed that they might require subsequent surgery to remove the plate. Future research should identify which factors are associated with hardware removal.

In this study, we compared patients with UIS based on etiology. In line with de Runz et al., we found a larger ulna positive variance in patients with secondary UIS than in patients with primary UIS [42]. Despite these differences between the subgroups before surgery, we did not find differences in postoperative patient-reported pain and function. This was also previously reported by Nunez et al. [43]. Based on our findings, there is no need to inform patients differently based on the etiology of UIS regarding potential pain relief and gain of function after USO.

It should be noted that the patients in this study who underwent USO for UIS secondary to a distal radius malunion did not have considerable angulation in the distal radius. Patients with a clinically relevant radial head displacement undergo corrective osteotomy of the distal radius in our clinics. This in is line with other institutions who recommend a corrective osteotomy of the distal radius instead of USO in case of 10° palmar inclination or  > 20° dorsal inclination from the normal tilt [9, 33, 44]. Stirling et al. investigated the patient-reported outcome following corrective osteotomy of the distal radius and also reported favorable results [45]. For patients with severe concomitant wrist instability, other treatment modalities may be necessary; however, this was outside the scope of this study.

This study involved a relatively large number of patients with UIS who underwent USO, evaluated using a standardized set of prospectively collected patient-reported and clinician-reported outcome measures. The routinely collected data provide valuable insights into the performance of the USO of our daily practice. Also, this study reflects the results from multiple surgeons performing diaphyseal oblique USO, which makes the outcomes more generalizable. We found beneficial outcomes in patients with primary UIS or secondary to distal radius malunion; however, patients should be informed that plate removal is often required and residual complaints might remain.

## Supplementary Information

**Additional file 1: Table S1. **Postoperative therapeutic regime after ulna shortening osteotomy.

**Additional file 2: Table S2. **ICHOM Complications in Hand and Wrist conditions (ICHAW), modified and derived from Clavien-Dindo 2009.

**Additional file 3: Table S3. **Differences in demographic data and patient-reported outcomes between responders and non-responders.

## Data Availability

The datasets used and/or analyzed during the current study are available from the corresponding author on reasonable request.

## Abbreviations

ECU: Extensor carpi ulnaris
ICHAW: International Consortium for Health Outcome Measurement Complications in Hand and Wrist conditions
IQR: Interquartile range
MCID: Minimal clinically important difference
MRI: Magnetic resonance imaging
NSAID: Nonsteroidal antiinflammatory drug
PROM: Patient-reported outcome measure
PRWHE: Patient rated wrist/hand evaluation
ROM: Range of motion
SD: Standard deviation
STROBE: Strengthening the Reporting of Observational Studies in Epidemiology
TFCC: Triangular fibrocartilage complex
UIS: Ulnar impaction syndrome
USO: Ulna shortening osteotomy
